# Region-based progressive localization of cell nuclei in microscopic images with data adaptive modeling

**DOI:** 10.1186/1471-2105-14-173

**Published:** 2013-06-02

**Authors:** Yang Song, Weidong Cai, Heng Huang, Yue Wang, David Dagan Feng, Mei Chen

**Affiliations:** 1 Biomedical and Multimedia Information Technology (BMIT) Research Group, School of Information Technologies, University of Sydney, NSW 2006, Australia; 2 Department of Computer Science and Engineering, University of Texas, Arlington, TX 76019, USA; 3 Bradley Department of Electrical and Computer Engineering, Virginia Polytechnic Institute and State University, Arlington, VA 22203, USA; 4 Intel Science and Technology Center on Embedded Computing, Carnegie Mellon University, Pittsburgh, PA 15213, USA

## Abstract

**Background:**

Segmenting cell nuclei in microscopic images has become one of the most important routines in modern biological applications. With the vast amount of data, automatic localization, i.e. detection and segmentation, of cell nuclei is highly desirable compared to time-consuming manual processes. However, automated segmentation is challenging due to large intensity inhomogeneities in the cell nuclei and the background.

**Results:**

We present a new method for automated progressive localization of cell nuclei using data-adaptive models that can better handle the inhomogeneity problem. We perform localization in a three-stage approach: first identify all interest regions with contrast-enhanced salient region detection, then process the clusters to identify true cell nuclei with probability estimation via feature-distance profiles of reference regions, and finally refine the contours of detected regions with regional contrast-based graphical model. The proposed region-based progressive localization (RPL) method is evaluated on three different datasets, with the first two containing grayscale images, and the third one comprising of color images with cytoplasm in addition to cell nuclei. We demonstrate performance improvement over the state-of-the-art. For example, compared to the second best approach, on the first dataset, our method achieves 2.8 and 3.7 reduction in Hausdorff distance and false negatives; on the second dataset that has larger intensity inhomogeneity, our method achieves 5% increase in Dice coefficient and Rand index; on the third dataset, our method achieves 4% increase in object-level accuracy.

**Conclusions:**

To tackle the intensity inhomogeneities in cell nuclei and background, a region-based progressive localization method is proposed for cell nuclei localization in fluorescence microscopy images. The RPL method is demonstrated highly effective on three different public datasets, with on average 3.5% and 7% improvement of region- and contour-based segmentation performance over the state-of-the-art.

## Background

Microscopic image analysis is becoming an enabling technology for modern systems-biology research, and cell nucleus segmentation is often the first step in the pipeline. Despite recent advances, the segmentation performance remains unsatisfactory in many cases. For example, on the popular public databases [1], the state-of-the-art segmentation accuracies are just around 85%.

**Figure 1:**
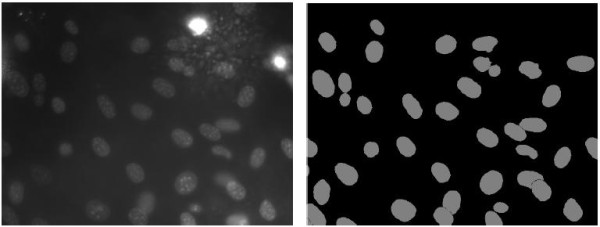
**An example image (left) and corresponding segmentation ground truth (right) from data set **[1]**.** It can be seen that besides the pixel-wise inhomogeneity within a cell nucleus, some cell nuclei exhibit much lower intensities than the others; and although the background looks generally dark, it is indeed highly inhomogeneous, with some fairly bright areas and also a few noisy regions displaying very high intensities.

The challenges of automated cell nucleus segmentation mainly arise from two imaging artifacts, as shown in Figure 1. First, the cell nuclei regions are inhomogeneous – the pixels of a cell nucleus exhibit non-uniform intensities and different cell nuclei also display varying patterns. Second, the background is also inhomogeneous and certain regions might have very similar appearance to the cell nuclei. These problems imply that: (1) precise delineation of boundaries between cell nuclei and the background is difficult; (2) some background areas could be mistaken as cell nuclei; and (3) certain cell nuclei could be missed. The segmentation problem can be characterized as a localization issue that includes both object detection and pixel-wise segmentation.

### Related work

Numerous works have been conducted on segmenting various structures in cell images [2,3], and unsupervised approaches appear to dominate. For example, the morphological methods based on thresholding, *k*-means clustering or watershed [4-8] can be quite effective, as long as the objects exhibit good contrast with the background. Watershed methods are also effective in separating touching cells, although the results might deviate from the actual contours slightly. A more popular trend of unsupervised segmentation is the energy-based deformable models, based on active contours [9] or level sets [10-15]. Compared with modeling contours explicitly, level sets have the advantage of being non-parametric and free from topology constraints. It is also relatively easy to incorporate continuous object-level regularization into level sets, such as shape priors. Another type of energy-based model is based on graph search [16,17], graph cuts [18,19] or normalized cuts [20]. Such methods attempt to derive the segmentation with global constraints, using well-defined graphical structures to represent the spatial relationships between regions. Many of these methods require good initial seeds or contours. However, the usual initialization techniques, such as thresholding and watershed, would not handle images with high inhomogeneities well, hence causing extra or missing detection of cell regions. Such detection errors during initialization could propagate into the final segmentation outputs.

It has been shown that intensity inhomogeneities can be tackled by integrating convex Bayesian functional with the Chan-Vese model [14], and discrete region-competition [15] based on the piecewise-smooth Mumford-Shah model [>[21]. However, without performing cell detection explicitly, the deformable models might become very complicated in order to filter background regions with cell-like features while keeping cell regions with background-like features. To detect cells from inhomogeneous background, one way is to reconstruct the ideal image [22,23], which however, requires specific imaging modeling. Reconstruction can also be built into active contours with constrained iterative deconvolution without explicitly computing the inverse problem [24,25]; however, it requires the point-spread function of a microscope, which is measured or modeled. Another way is to enhance the objects using h-dome transformation [26]; however, it might have difficulties with inhomogeneous foreground. The inhomogeneity can also be reduced with reference-based intensity normalization [27]; however, the image-level normalization would not well handle the intra-image variation. In addition, shape-based nucleus detection has been proposed, with Laplacian of Gaussian (LoG) [28] or sliding band filter (SBF) [29]. While the latter method is less sensitive to low contrast and better representative of irregular shapes, the detection accuracy partially relies on validation from the corresponding cytoplasm image, which is not always available.

Different from the unsupervised approaches, classification-based methods have also been proposed to incorporate prior information from labeled images. These classifiers include Bayesian [30], *k*-nearest neighbor (*k*NN) [31], support vector machine (SVM) [32], and atlas-based approaches [33]. Since the apparent difference between the foreground and background is their intensities, simple intensity-based features, such as histograms [30], have been widely used. On the other hand, the effectiveness of classification-based methods depends highly on the separation of feature spaces between foreground and background. Therefore, approaches based on a bag of local classifiers [30], and more complex features such as the local Fourier transform (LFT) [31], spatial information [33], and combination of appearance, shape and context features [32], have been proposed.

While most such supervised approaches describe the pixel- or region-level features, there are methods that tackle intensity inhomogeneity by explicitly modeling the inter-cell variations as more structural features. One way is to perform color standardization within pixelwise classification [34] to account for the inter-image intensity variations. To also address the intra-image variations, contrast information between an image region and the global foreground and local interest regions is computed [35]. A similar approach is to estimate foreground probabilities based on intensity distributions derived from global images and local detection outputs [36]. While both approaches introduce cell-adaptive features, the methods for global feature representation and local region detection might not work well with large feature overlapping between the foreground and the background. An additional false positive reduction step has also been proposed to remove bright background regions that are misidentified as cell nuclei [37]. However, this approach requires a learned classifier, whose performance could be affected by inter-image feature variation. More differently, registration-based approach has also been studied, by creating a template set from training images and segmenting the testing image based on best matches [38]; such templates however, might have difficulties capturing large varieties of object shapes and textures.

### Our contribution

**Figure 2:**
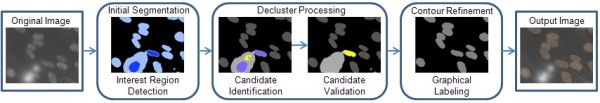
**The high-level flow chart of our proposed region-based progressive localization method.** In this example, the cell nuclei and clusters are first extracted during initial segmentation with contrast-enhanced salient region detector, then missed or falsely detected cell nuclei are further processed in decluster processing with classification-based candidate identification and probability estimation via distance profile for candidate validation, and better contour delineation is finally achieved with regional contrast-based graphical model. For easier viewing, a quadrant of the original image is shown here, and similarly for Figure 3 and 4. The meaning of the color coding is described in Figures  3, 4 and 5.

The contribution of our work is to localize the cell nuclei in images with high intensity inhomogeneity with various data-adaptive modeling techniques in a progressive manner. Specifically, we design a three-stage cell nucleus localization method that: (1) salient regions representing cell nuclei and cell clusters are extracted with image-adaptive contrast enhancement; (2) the clusters are further processed to identify true cell nuclei based on feature-distance profiles of reference regions with cluster-adaptive probability estimates; and (3) the contours of detected cell nuclei are refined in a graphical model with region-adaptive contrast information. Figure 2 gives an overview of the proposed method.

We also design distinctive data-adaptive priors that can be categorized by the level of generalization: (1) global-level features modeled as support vectors from training images; (2) image-level features representing the distribution of varying appearances of the nearby cell nuclei; and (3) region-level features computed at all three stages of the method for interest region detection, candidate validation and contour refinement. Being adaptive to the specific image or interest region, the image- and region-level features are especially effective in accommodating the intensity inhomogeneities.

Compared to localization methods based on global criteria (e.g. thresholding or feature-based classification), our approach is more capable of accommodating (1) intensity variations between cell nuclei (intra- and inter-images) and (2) feature overlapping between cell nuclei and background areas. Compared to the energy-based techniques that target pixel-wise segmentation (e.g. level sets and graph cuts), our method has a stronger focus on cell nuclei detection with explicit modeling of cell-specific characteristics, to effectively filter cell-like background regions and identify obscure cell nuclei.

We suggest that the proposed region-based progressive localization (RPL) method can be potentially extended to other localization problems, if the objects of interest can be modeled as regions with distinct features from the surrounding background. A similar three-stage approach would be used, and the application-specific modifications would mainly focus on the feature design. One example could be tumor localization in functional images.

## Methods

### Initial segmentation

While cell nuclei might appear similar to the background, there is always some degree of contrast between them. Such an observation motivates us to localize the cell nuclei by extracting salient regions. During initial segmentation, we do not have strict requirements about the extracted regions. In particular, if multiple cell nuclei are tightly connected, or cell nuclei are surrounded by high-intensity background and difficult to differentiate, identifying them as a single region is acceptable. We design a *contrast-enhanced salient region detector* for initial segmentation.

**Figure 3:**
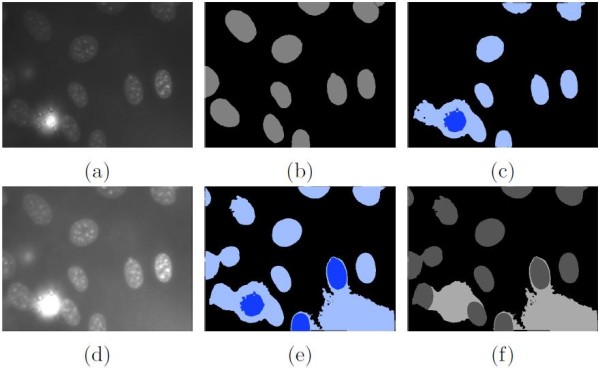
**Illustration of initial segmentation.(a)** The original image. **(b)** The segmentation ground truth. **(c)** The interest regions detected without iterative contrast enhancement, and darker blue denotes upper-level regions. **(d)** The image after iterative enhancement. **(e)** The final interest regions detected. After the initial segmentation, decluster processing is performed, with outputs shown in **(f)** and dark gray indicating the detected cell nuclei.

Specifically, an iterative approach is developed based on the maximally stable extremal region (MSER) method [39]. Since MSER does not require any initial contour and the region stability is constrained by local regional information, it is easy to use and able to accommodate large intra-image variations. However, the effectiveness of MSER depends highly on the intensity contrast between the foreground and background. If the contrast is low, some regions would not be detected (e.g. Figure 3c). It is intuitive to add contrast enhancement. However, basic approaches such as intensity stretching would not work due to large intensity span. Instead, we design an iterative approach by alternating between the following two steps. First, interest regions {*R*} are detected using MSER, as shown in Figure 3c. Second, based on the detection result, the image is enhanced (Figure 3d) by:

(1) $$I:=\frac{I}{0.5({\left\{R\right\}}_{0}+{\left\{R\right\}}_{2})}\cdot C$$

where {*R*}_0_ and {*R*}_2_ denote the minimum and mean intensities of the detected interest regions in *I*, and *C* is a scaling constant. The normalization factor 0.5({*R*}_0_+{*R*}_2_) is chosen based on: (1) it should normally be smaller than *C* so that all pixels in *I* are scaled up with contrast between pixels increased proportionally; and (2) it should not be so small that the image becomes distorted from the original patterns with intensities capped at 255 for grayscale images. The iteration stops when the number of regions created does not change any further. With such a contrast-enhanced approach, better region detection output can be seen in Figure 3e. The resultant regions are either single-level, or form a hierarchy of lower- and upper-level regions.

It is also observed that during each iteration, the parameter *MaxVariation* in MSER (using VLFeat library [40]) needs to vary for individual images to better accommodate the inter-image variations. Therefore, the parameter value is determined at runtime by first setting *MaxVariation* to *v*_1_ then gradually reducing it by a certain step *Δ*_*v*_ until it reaches *v*_2_ or the number of region levels is larger than one. Furthermore, while the resultant single- and upper-level regions are mostly cell nuclei, occasionally under-segmentation happens. In other words, a single cell nucleus could be divided into two nested regions and the upper-level region would become a under-segmented portion of the cell nucleus. To reduce such under-segmentation, we find that if the combined area of two nested regions is roughly elliptical with a suitable size, they can be merged as a single region. The shape and size criteria are determined using a linear-kernel binary SVM obtained from the training data. The overall process of initial segmentation is listed in Algorithm 1.

#### **Algorithm 1: Initial segmentation**

### Decluster processing

As seen in Figure 3e, the detected single- and upper-level regions usually represent the cell nuclei, and lower-level regions usually represent the background with elevated intensities. However, the upper-level regions could contain false positives caused by bright background, and lower-level regions could also include undetected cell nuclei. It is also observed that such incorrect detections are mainly present among the two-level nested regions (i.e. clusters), while the single-level regions are normally true cell nuclei. Therefore, in the second stage, we focus on further processing on the detected clusters, with two objectives. First, we expect to identify any cell nucleus that has not been detected after the initial segmentation. Such cell nuclei typically exhibit similar intensities to the surrounding background, and hence would not be highlighted as salient regions. Second, we need to filter out high-intensity background regions, which usually have rounded or irregular shapes, and could be easily confused as cell nuclei. A two-step approach is designed, using candidate identification then candidate validation. An example output is shown in Figure 3f.

Formally, let *U* = {*u*_*i*_ : *i* = 1,...,*N*_*U*_} be a detected cluster, with *N*_*U*_ pixels *u*_*i*_. Define the set of labels {*F*,*B*} representing the foreground (i.e. cell nuclei) and background respectively, and a foreground region as a connected component *G*_*x*_ ⊂ *U* with ∀*u*_*i*_ ∈ *G*_*x*_ : *l*_*i*_=*F*. The problem is to label each pixel *u*_*i*_∈*U* as *l*_*i*_ = {*F*,*B*}, with the object-level constraint that any detected foreground region *G*_*x*_ should have suitable characteristics as a cell nucleus.

#### ***Candidate identification***

In the first step, we try to identify a set of non-overlapping candidate foreground regions {*G*_*x*_} from each cluster *U* by labeling each pixel *u*_*i*_ as foreground or background. We specify that any upper-level region enclosed in a cluster *U* is a candidate region *G*_*x*_. To identify more candidates from the cluster *U* itself, it is observed that to differentiate between *F* and *B* pixels, the texture feature in a local patch is more discriminative than pixel intensities. For example, compared with cell nuclei, the background usually has more homogeneous texture that might be dark or bright. In this work, we choose to use the scale-invariant feature transform (SIFT) descriptor [41], which describes the gradient distribution within a local patch and is invariant to scale, translation and rotation. SIFT feature of each pixel *u*_*i*_ is computed, and then labeled using a binary SVM. The SVM kernel is polynomial, with other default settings in LIBSVM [42]. A connected component of *F* pixels is identified as a candidate region *G*_*x*_.

**Figure 4:**
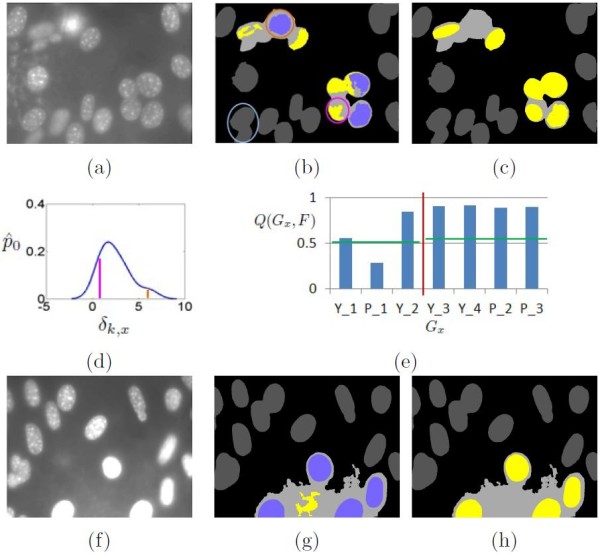
**Illustration of decluster processing.(a)** The example image (after iterative enhancement). **(b)** Newly identified candidate regions are shown in yellow, with purple indicating the ones detected during initial segmentation, and both gray and purple denoting the reference regions; here to illustrate the probability inference, the light blue circle highlights one reference *G*_*k*_, and pink and orange circles indicate two candidate regions *G*_*x*_. **(c)** The candidates validated shown as yellow. **(d)** The KDE plot generated for *G*_*k*_, in which the pink and orange lines represent ${\hat{p}}_{0}(\delta_{k,x})$ for the pink and orange circled candidates. **(e)** The probabilities *Q*(*G*_*x*_,*F*) derived for all candidate regions, with P_1 and Y_4 corresponding to the orange and pink circled candidate regions, the red vertical line separating the two clusters, and green horizontal lines indicating the thresholds for *l*(*G*_*x*_) = *F*. **(f)**-**(h)**: A second example with the same annotations to show that different from the first example, the real cell nuclei here are bright while the filtered candidate region is darker.

While most of the candidate regions are true cell nuclei, some are actually bright background areas with round shapes (e.g. the first example in Figure 4). To filter out the false detections, we pass them to the candidate validation stage.

#### ***Candidate validation***

In the second step, we validate if the identified candidate region *G*_*x*_ in image *I* is a cell nucleus. There are two reasons that motivate this step. First, there might be misclassification during candidate identification due to inter-image intensity variations (e.g. different appearances of the cell nuclei between the two examples in Figure 4). The labeling performance could be improved based on reference information gathered from the testing image itself. Second, pixel-level labeling based on SIFT features has limited spatial information and often does not represent the overall region *G*_*x*_. We design a *probability estimation via distance profile* method to derive the probability *Q*(*G*_*x*_,*F*) of *G*_*x*_ being a valid cell nucleus based on the feature-distance profiles of other reference cell nuclei, as detailed below.

##### 

**Probability inference** Although cell nuclei in an image could have varying characteristics, we expect that *G*_*x*_, if representing a true cell nucleus, should have similar features to the other cell nuclei in the same image, especially those spatially adjacent to *G*_*x*_, as can be seen from the examples in Figure 4. Therefore, if we have a set of determined cell nuclei in *I*, we can use them as references to validate *G*_*x*_. To cope with inter-image variations, we would only select references from the image *I* in which *G*_*x*_ resides. This means we could not use the ground truths for reference construction. Instead, we use the single- and upper-level regions that are detected during initial segmentation as references.

We use these references by first creating a distance profile per reference, and computing the probability of *G*_*x*_ being a cell nucleus based on its feature distance to each reference. Specifically, assume within an area near *G*_*x*_, there are *K* reference regions $G=\{G_{k}:k=1,\mathrm{...},K\}$. Here *near* is defined as both *G*_*x*_ and *G*_*k*_ being in the same quadrant of image *I*. Let *f*_*x*_ describe the region-level feature of *G*_*x*_, and the feature distance between *G*_*x*_ and *G*_*k*_ as *δ*(*f*_*k*_,*f*_*x*_) (details of *f* and *δ* in the next two subsections). Intuitively, the more similar *G*_*x*_ and *G*_*k*_ are, the more likely *G*_*x*_ is a cell nucleus. However, since *G*_*k*_ may be a false positive detection, decision based on direct feature distance *δ*(*f*_*k*_,*f*_*x*_) might be error prone. Therefore, we devise an alternative hypothesis that, if *δ*(*f*_*k*_,*f*_*x*_) is comparable with $\left\{k^{\prime }=1,\mathrm{...},K,k^{\prime }\neq k:\delta (f_{k},f_{k^{\prime }})\right\}$, then *G*_*x*_ is likely a cell nucleus.

To measure if *δ*(*f*_*k*_,*f*_*x*_) is comparable with $\left\{\forall k^{\prime }:\delta (f_{k},f_{k^{\prime }})\right\}$, we use the non-parametric kernel density estimation (KDE):

(2) $${\hat{p}}_{0}(\delta_{k,x})=\frac{1}{K-1}\underset{k^{\prime }\neq k}{\sum }\frac{1}{h_{k}}K(\frac{\delta_{k,x}-\delta_{k,k^{\prime }}}{h_{k}})$$

where *δ*_*k*,*x*_ is short for *δ*(*f*_*k*_,*f*_*x*_), K(·) is the Gaussian kernel and *h*_*k*_ is the bandwidth approximation following normal distribution of all data samples $\left\{\forall k^{\prime }:\delta (f_{k},f_{k^{\prime }})\right\}$. The density value ${\hat{p}}_{0}(\delta_{k,x})$ is then normalized by the maximum density of the distribution to obtain the comparability measure in terms of probability $\hat{p}(\delta_{k,x})\in [0,1]$:

(3) $$\hat{p}(\delta_{k,x})={\hat{p}}_{0}(\delta_{k,x})/\underset{k^{\prime }}{\max}\{{\hat{p}}_{0}(\delta_{k,k^{\prime }})\}$$

With this model, $\hat{p}(\delta_{k,x})$ is larger when *δ*_*k*,*x*_ approaches the Gaussian mean of the samples, which means that *G*_*x*_ is more likely a cell nucleus if the distance between *G*_*x*_ and *G*_*k*_ is similar to how the other references $\left\{G_{k^{\prime }}\right\}$ vary from *G*_*k*_.

Next, by combining the estimates $\hat{p}(\delta_{k,x})$ from all references {*G*_*k*_}, the final probability of *G*_*x*_ being a cell nucleus is derived:

(4) $$Q(G_{x},F)=\frac{1}{K}\underset{k}{\sum }\hat{p}(\delta_{k,x})$$

The averaging operation helps to ensure that a single reference *G*_*k*_ with very different features from *G*_*x*_ would not affect the overall probability *Q*(*G*_*x*_,*F*) significantly.

Then, based on *Q*(*G*_*x*_,*F*), we define a thresholding rule to determine if *G*_*x*_ is a valid cell nucleus:

(5) $$l(G_{x})=F\text{, if}\left\{\begin{array}{c} Q(G_{x},F)>\alpha_{1}\underset{x^{\prime }}{\max}Q(G_{x^{\prime }},F),\\ Q(G_{x},F)>\alpha_{2}\text{, for}G_{\mathrm{x\prime}}=\emptyset \end{array}\right)$$

where $G_{x^{\prime }}$ denotes other candidate regions that are within the same cluster *U* as *G*_*x*_ and *x*^**′**^ ≠ *x*; *α*_1_ and *α*_2_ are predefined thresholds. Examples of the density computation and probability derivation are shown in Figure 4, and the overall process of candidate validation is listed in Algorithm 2.

###### **Algorithm 2: Candidate validation**

##### 

**Appearance feature** We observe that a region tends to comprise patches of similar textures and repetitive patterns. Therefore, we choose to represent *G*_*x*_ with bag-of-features. First, the image *I* that contains *G*_*x*_ is divided into a grid of patches {*P*}. Then for each patch, we represent its texture feature by its minimum, maximum, mean intensity, standard deviation, and a histogram of intensity differences between each pair of pixels. Each patch-wise feature is then assigned a feature word. A histogram summarizing the occurrence frequencies of such feature words in *G*_*x*_ is defined as *f*_*x*_. Here each feature vector is normalized by the size of *G*_*x*_, to represent the percentages of various intensities and feature words in *G*_*x*_.

Note that if *G*_*x*_ is a newly identified candidate during decluster processing, *G*_*x*_ might only represent a small under-segmented portion of the actual cell nucleus due to the pixel-level labeling. Therefore, to have a good summary of the actual candidate feature, we first estimate an elliptical region $G_{x^{o}}$ that is a minimum volume ellipsoid covering *G*_*x*_[43]. To avoid including many background pixels into $G_{x^{o}}$, we ensure $G_{x^{o}}$ is part of the cluster *U* in which *G*_*x*_ is detected: $G_{x^{o}}=G_{x^{o}}\cap U$. $G_{x^{o}}$ is then used in place of *G*_*x*_ as the detected candidate, from which *f*_*x*_ is computed. The refined elliptical regions are shown in Figure 4c.

##### 

**Appearance distance** To compute the distances *δ*(*f*_*k*_,*f*_*x*_) between two histogram features, the diffusion distance [44] is used. The diffusion distance models the distance between histogram-based descriptors as heat diffusion process on a temperature field. Compared to the bin-to-bin histogram distances, such as Euclidean distance, the diffusion distance is able to measure cross-bin distances, avoiding explicit computation of histogram alignment. While the earth mover’s distance (EMD) [45] has similar advantages, the computation of diffusion distance is much faster, with *O*(*H*) complexity only, where *H* is the number of histogram bins.

### Contour refinement

At this stage, a detected region could contain a single or multiple cell nuclei, which could be under-segmented or include extra background. We thus expect to achieve better contour delineation of cell nuclei. Our idea is that, while the foreground and background are often inhomogeneous, there is always relatively good contrast between them in a local area. Therefore, by performing contour refinement for each detected cell region *G* individually, the foreground and background can be better differentiated by analyzing the localized contrast information. We employ a *regional contrast-based graphical model* for the contour refinement.

**Figure 5:**
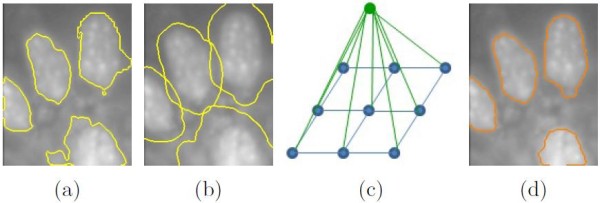
**Illustration of contour refinement.(a)** Segmentation output after the decluster processing shown with yellow contours. **(b)**$\bar{G}$ indicated with yellow contours. **(c)** Visualization of the graphical model, with blue nodes representing *l*_*i*_ and green node *l*_*G*_, and the blue and green edges denoting the pairwise relationships. **(d)** Results of contour refinement with orange contours.

Specifically, a conditional random field (CRF) [46] with the following energy function is designed:

(6) $$\begin{array}{lll} E(L|\bar{G})=\underset{i}{\sum }\eta (l_{i}) & +\eta (l_{G})+\;0.5\{\underset{i}{\sum }\varphi (l_{i},l_{G})\; & \\ & +\underset{i,i^{\prime }}{\sum }\phi (l_{i},l_{i^{\prime }})\}\; \end{array}$$

where $\bar{G}$ denotes the detected region *G*  plus its surrounding area of a fixed width (half of the short axis of *G*) (Figure 5b), and *L* denotes the labeling vector of all pixels in $\bar{G}$. Then, the model attempts to refine the contour of *G* by relabeling each pixel $u_{i}\in \bar{G}$ as *l*_*i*_ = {*F*,*B*}. Here *η*(*l*_*i*_) is the unary contrast-based intensity term, *η*(*l*_*G*_) combined with *φ*(·) is the contrast-based detection term with *l*_*G*_ representing the detected region *G*, and *ϕ*(·) is the spatial term associating neighboring pixels *u*_*i*_ and $u_{i^{\prime }}$. The constant 0.5 is set to obtain equal contributions from the unary costs ($\underset{i}{\sum }\eta (l_{i})+\eta (l_{G})$) and the combined pairwise costs ($\underset{i}{\sum }\varphi (l_{i},l_{G})+\underset{i,i^{\prime }}{\sum }\phi (l_{i},l_{i^{\prime }})$). Graph cut [47] method is used to derive the most probable labeling *L* that minimizes the energy function, to produce the final segmentation of cell nuclei from $\bar{G}$. Here our customized definition of the intensity term and inclusion of the detection term are the main distinctions from the other CRF constructs [35,48,49].

The contrast-based intensity term *η*(*l*_*i*_) describes the unary costs of pixel *u*_*i*_ labeled as *l*_*i*_ ∈ {*F*,*B*}. Basically, the costs of *l*_*i*_ = *F* and *l*_*i*_ = *B* represent the inverse probabilities, and the probability *pr*(*u*_*i*_,*F*) of *l*_*i*_ = *F* is computed by:

(7) $$\begin{array}{ll} \text{pr}(u_{i},F) & ={\left(1+\text{exp}(-2(f_{i}-\lambda_{G}))\right)}^{-1}\; \end{array}$$

(8) $$\begin{array}{ll} f_{i} & =I_{i}/I_{G}\; \end{array}$$

(9) $$\begin{array}{ll} \lambda_{G} & =f_{G}-\gamma_{G}(f_{G}-⌞f_{G})\; \end{array}$$

where *I*_*G*_ denotes the mean intensity of *G*, and *pr*(*u*_*i*_,*F*) follows a sigmoid probability distribution based on the contrast feature *f*_*i*_. We expect pixels with *f*_*i*_ > *λ*_*G*_ to more likely represent the foreground. *λ*_*G*_ is computed based on *f*_*G*_ and $⌞f_{G}$, which are the mean and minimum of all feature values {*f*_*i*_ : *u*_*i*_ ∈ *G*}, and is adjusted by *γ*_*G*_ for a balance of foreground and background partitioning in *G*. The parameter *γ*_*G*_ is calculated at runtime, by gradually increasing it from *γ*_1_ to *γ*_2_ with a step value *Δ*_*γ*_, and choosing the smallest *γ*_*G*_ ∈ [ *γ*_1_,*γ*_2_] that does not cause the entire *G* to be labeled as *B*. With *pr*(*u*_*i*_,*B*) = 1 - *pr*(*u*_*i*_,*F*), the cost values for both labels are:

(10) $$\eta (l_{i})=1-\text{pr}(u_{i},l_{i})$$

Note that since *λ*_*G*_ would be closer to *f*_*G*_ in most cases with small *γ*_*G*_, it would cause portions of *G* to have *pr*(*u*_*i*_,*F*) < 0.5 (i.e. *u*_*i*_ labeled as background), resulting in possible under-segmentation. It is however not advisable to lower *λ*_*G*_, due to considerable overlap between low-intensity areas in *G* and the background. Therefore, we introduce a second contrast-based detection term to encourage labeling of *l*_*i*_ = *F*. An auxiliary node *l*_*G*_ is first included to the graph with the following unary costs:

(11) $$\eta (l_{G})=\left\{\begin{array}{cc} 0 & \;\text{if}\;l_{G}=F\\ N_{\bar{G}} & \;\text{otherwise} \end{array}\right)$$

where $N_{\bar{G}}$ is the number of pixels in $\bar{G}$, and such a large cost of *l*_*G*_ = *B* ensures *l*_*G*_ is assigned 1. Then for each pixel *u*_*i*_, a pairwise cost *φ*(*l*_*i*_,*l*_*G*_) is computed based on the contrast *ν*(*I*_*i*_,*I*_*G*_) between *I*_*i*_ and the mean intensity of *G*:

(12) $$\varphi (l_{i},l_{G})=\delta (l_{i}-l_{G})\cdot \nu (I_{i},I_{G})$$

with *δ*(*l*_*i*_ - *l*_*G*_) = 1 if *l*_*i*_ ≠ *l*_*G*_ and 0 otherwise, and *ν*(*I*_*i*_,*I*_*G*_) = 1 if *I*_*i*_ > *I*_*G*_, or:

(13) $$\nu (I_{i},I_{G})=\text{exp}(-\frac{||I_{i}-I_{G}|{|}^{2}}{2\langle ||I_{i}-I_{G}|{|}^{2}\rangle })$$

where 〈 · 〉 denotes the average Euclidean distances of all such pairwise distances in $\bar{G}$. In this way, pixels with *pr*(*u*_*i*_,*F*) ≈ 0.5 could be better labeled with the additional cost factor; and obvious background pixels would still obtain the correct *B* label, with *φ*(*l*_*i*_,*l*_*G*_) much lower than *η*(*l*_*i*_).

The spatial term $\phi (l_{i},l_{i^{\prime }})$ then further enhances the delineation by encouraging spatial labeling consistencies between neighboring pixels *u*_*i*_ and $u_{i^{\prime }}$. A pairwise cost for $l_{i}\neq l_{i^{\prime }}$ is thus defined as:

(14) $$\phi (l_{i},l_{i^{\prime }})=\delta (l_{i}-l_{i^{\prime }})\cdot \nu (I_{i},I_{i^{\prime }})$$

where *δ*(·) and *ν*(·) follow Eq. (12). Such a cost function implies that pixels with more similar intensities would be more penalized if they take different labels.

### Materials and evaluation methods

**Figure 6:**
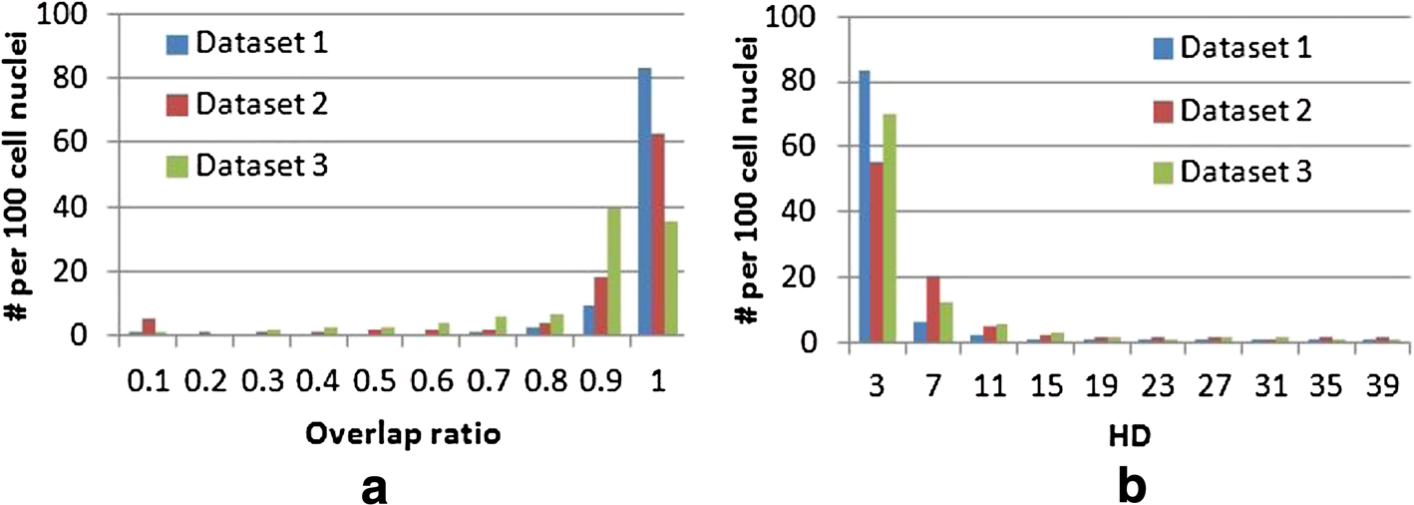
**Example localization results.** The top row from dataset 1, the middle row from dataset 2 and the bottom row from dataset 3. **(a)** Image with ground truth contours in orange. **(b)** Results using our proposed RPL method. **(c)** Results using OT. **(d)** Results using LS.

Three different datasets that are publicly available with segmentation ground truth are used in this study. Their main properties are summarized in Table 1. The images in the first two datasets were acquired with nuclear markers whereas the third dataset also includes the cytoplasm. Detailed information can be found in [1,50]. Among the three, dataset 1 has higher contrast between cell nuclei and background. Datasets 2 and 3 have large intensity inhomogeneity and considerable degree of intensity overlapping between the cell nuclei and the background. The inclusion of cytoplasm in dataset 3 poses more challenges. The images in dataset 3 are preprocessed to remove the pink areas and converted to grayscale. Figure 6 shows an example image after the preprocessing.

**Table 1:** Summary of the datasets used

	**Dataset 1**	**Dataset 2**	**Dataset 3**
	[1]	[1]	[50]
Cell type	U2OS	NIH3T3	Serous
# images	48	49	10
# cells	1831	2178	254
Image size	1349 ×1030	1344 ×1024	512 ×512
Color map	Grayscale	Grayscale	RGB
Structure	Nuclei	Nuclei	Nuclei & Cytoplasm

Most parameters used in this study are set to the same values for all three datasets: (1) in *Initial Segmentation*, *v*1 = 0.7, *v*2 = 0.4 and *Δ*_*v*_ = 0.1; (2) in *Probability Inference*, *α*_1_ = 0.6 and *α*_2_ = 0.4; (3) in *Appearance Feature*, the number of histogram bins is 64, and the number of feature words is 12; and (4) in *Contour Refinement*, *γ*_1_ = 0.25, *γ*_2_ = 1 and *Δ*_*γ*_ = 0.25. While these settings are chosen empirically, using a common setting for all three datasets suggests that the method is robust to different image acquisition and manual tuning of parameters can be minimal. There are only two dataset-specific parameters. One is the patch size in *Appearance Feature*, which is 8×8 pixels for datasets 1 and 2, and 4×4 pixels for dataset 3. The smaller size for dataset 3 is chosen due to its smaller cell nuclei compared to datasets 1 and 2. The other parameter is *C* in *Initial Segmentation*, which is set to 128 for datasets 1 and 2, and 64 for dataset 3. This ensures the contrast enhanced images in dataset 3 would not become too bright to cause distortion.

For dataset 1, four representative images are selected to train two SVM classifiers, for the cell-cluster differentiation and candidate identification. While testing is performed on all images to make the results directly comparable with the state-of-the-art [14,38], we note that the testing results are not sensitive to the selection of training data, with very similar testing results observed based on different training sets. Similar procedures are performed for dataset 2. For dataset 3, in order to have comparable performance evaluation with [32,35], half of the images are used for training (images # 2, 3, 4, 5, and 7) and the rest for testing.

We evaluate the localization of cell nuclei by two measures. First, performance of object-level detection is evaluated by recall (R), precision (P), and accuracy (A):

(15) $$\begin{array}{ll} R & =\text{TP}/(\text{TP}+\text{FN})\; \end{array}$$

(16) $$\begin{array}{ll} P & =\text{TP}/(\text{TP}+\text{FP})\; \end{array}$$

(17) $$\begin{array}{ll} A & =\text{TP}/(\text{TP}+\text{FN}+\text{FP})\; \end{array}$$

where TP, FN, and FP are the numbers of true positive, false negative and false positive detections of cell nuclei. Given a detected region *O*_*d*_ and the ground truth mask *O*_*gt*_, if the overlap ratio *R*(*O*_*d*_) is at least 0.5:

(18) $$R(O_{d})=|O_{d}\cap O_{\text{gt}}|/|O_{d}\cup O_{\text{gt}}|$$

then the detection is considered TP [51]; and correspondingly FN and FP are determined.

Second, the segmentation performance is evaluated by both region- and contour-based measures, including Dice, normalized sum of distances (NSD) and Hausdorff distance (HD):

(19) $$\begin{array}{ll} \text{Dice} & =2|F\cap M|/(|F|+|M|)\; \end{array}$$

(20) $$\begin{array}{ll} \text{NSD} & =\underset{u_{i}\in (F△M)}{\sum }D(u_{i})/\underset{u_{i}\in (F\cup M)}{\sum }D(u_{i})\; \end{array}$$

(21) $$\begin{array}{ll} \text{HD} & =\underset{u_{i}\in \mathrm{\partial F}}{\max}D(u_{i})\; \end{array}$$

Here *F* represents the foreground pixels identified, *M* is the ground truth mask, and *D*(*u*_*i*_) is the minimal Euclidean distance of pixel *u*_*i*_ to *∂M* of the corresponding reference nuclei, with *∂* indicating the contour.

We have compared with popular cell imaging segmentation techniques, including Otsu thresholding, *k*-means clustering and watershed [8]. Furthermore, in view of the popularity of level set for cell imaging and our design on tackling the intensity inhomogeneities, we have experimented with a level set method that has a similar focus, using the authors’ released code [52], with initial contours generated using watershed method. For all methods, post-processing is conducted to remove isolated segments that are smaller than 1/10 of the average size of foreground regions detected in the image. In addition, we report direct performance comparisons with the state-of-the-art results reported on the same datasets [14,32,35,38], by including the same performance measures as used in these works.

## Results and discussion

### Cell detection

**Figure 7:**
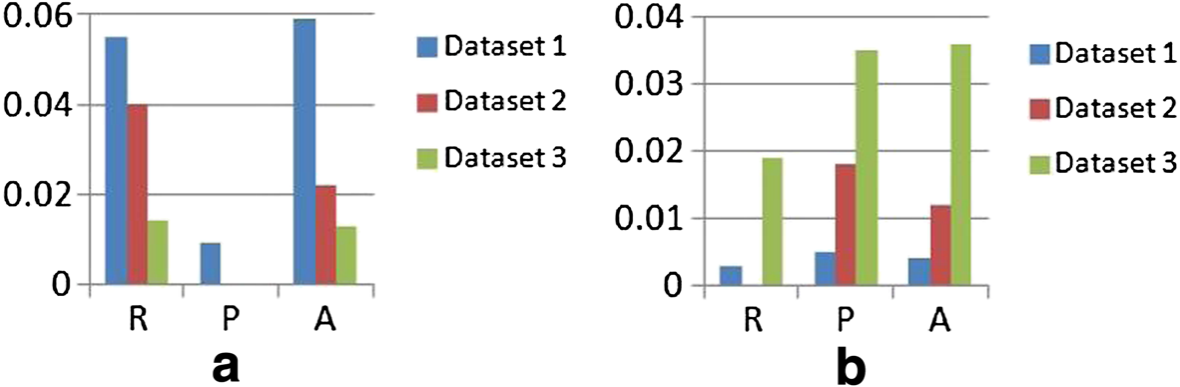
**Cell detection results.** Histograms with *y*-axis as numbers from per 100 cell nuclei, and *x*-axis as **(a)** the object-level overlap ratio and **(b)** the Hausdorff distance, both between the segmented foreground and ground truth.

We report the object-level detection results in Table 2. Comparing the results at various stages of the methodology, the improvement is larger on dataset 2 than dataset 1, e.g. 8.3% increase in detection accuracy on dataset 2 vs 0.8% increase on dataset 1. This is because inhomogeneity is more prominent on dataset 2 while dataset 1 exhibits clearer contrast between the cell nuclei and the background in most images. In our evaluation, a detection is only considered as TP if the overlap ratio in Eq. (18) is at least 0.5. Therefore, a largely over- or under-segmented object would be counted as FN for the second stage, and corrected after the contour refinement. This explains why although cell nuclei are detected after the decluster processing, the recall results only improve significantly after the third stage. On dataset 3, the presence of cytoplasm causes many cell nuclei to clutter into one region during the initial segmentation; this leads to FN. The third stage better differentiates the cell nuclei and cytoplasm, and the improvement is significant with 18.7% and 3.4% increase in detection recall and precision. Figure 7a gives a better overview of the overlap ratios obtained from the final localization outputs. While most cell nuclei exhibit ratios not less than 0.5, less optimal results are observed on dataset 3 again due to the influence from the cytoplasm.

**Table 2:** Detection results

	**Dataset 1**			**Dataset 2**			**Dataset 3**		
	**S-1**	**S-2**	**S-3**	**S-1**	**S-2**	**S-3**	**S-1**	**S-2**	**S-3**
R	0.967	0.973	0.975	0.814	0.834	0.886	0.734	0.770	0.957
P	0.929	0.935	0.940	0.933	0.941	0.955	0.915	0.941	0.975
A	0.908	0.914	0.916	0.769	0.792	0.852	0.691	0.736	0.934

After initial segmentation (S-1), decluster processing (S-2), and contour refinement (S-3).

**Figure 8:**
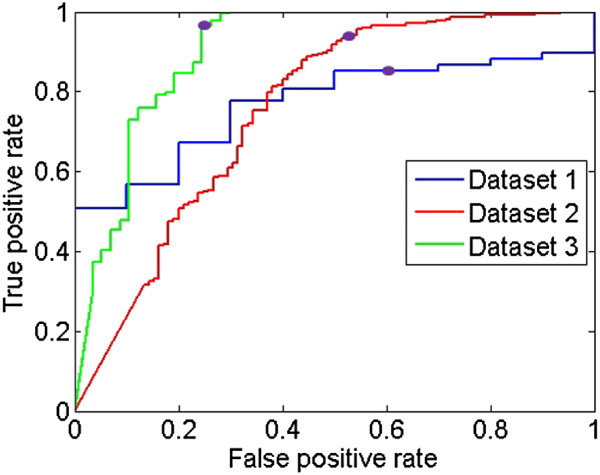
**Cell detection results.** Improvement on detection from **(a)** iterative image enhancement and interest-region extraction, and **(b)** candidate validation.

The performance improvement introduced by the iterative process of interleaving interest region extraction and image enhancement are shown in Figure 8a. The higher recall (i.e. on average 3.6% increase) suggests that such an approach is especially useful for identifying foreground regions that originally display low contrast from the background. The benefits of having candidate validation are shown in Figure 8b. By filtering out interest regions that are very different from the reference regions, the detection precision thus improves by on average 2%. The recall improves by on average 0.7% only, mainly because of the same constraints imposed by the overlap ratio.

**Figure 9:**
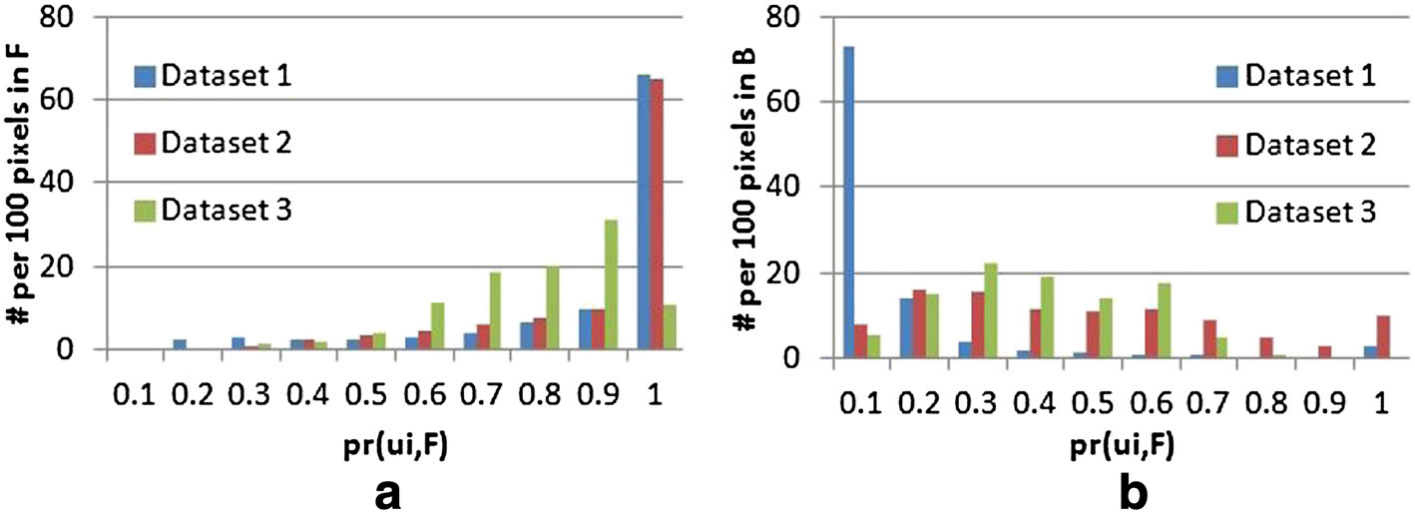
**ROC curves of candidate validation.** The purple dots indicate the position with the decision threshold *α*_1_ = 0.6.

To evaluate the effect of the default threshold setting *α*_1_ for candidate validation, the receiver operating characteristics (ROC) curves are plotted by varying the threshold value. The probability estimates $Q(G_{x},F)/\underset{x^{\prime }}{\max}Q(G_{x^{\prime }},F)$ from all candidate regions are included in the plot, and candidate regions with at least 0.5 overlapping ratio with the ground truth are marked as foreground class and the rest as the background class. As shown in Figure 9, the 0.6 threshold setting provides a good balance between the TP and FP detections, with close to maximum TP rates. Note that the numbers of true negatives here are small (about 1/5 of positive samples), hence the FP rates appear relatively high.

### Nucleus segmentation

Table 3 summarizes the region- and contour-based segmentation results. On datasets 2 and 3, the decluster processing improves the Dice measure by about 3% and 4%, due to better object-level labeling of candidate regions. The contour-based measures, however, are mainly enhanced at the third stage of the methodology, with on average more than half reduction in NSD and HD. This is attributed to better contour delineations based on the detection results from the first two stages. Besides the mean values listed in the table, the distributions of Hausdorff distances on the final localization results are also shown in Figure 7b.

**Table 3:** Segmentation results

	**Dataset 1**			**Dataset 2**			**Dataset 3**		
	**S-1**	**S-2**	**S-3**	**S-1**	**S-2**	**S-3**	**S-1**	**S-2**	**S-3**
Dice	0.948	0.954	0.958	0.847	0.876	0.906	0.815	0.853	0.924
NSD	0.026	0.023	0.017	0.175	0.141	0.090	0.338	0.327	0.052
HD	10.72	10.60	10.01	22.55	20.76	14.10	20.96	18.03	5.51

After initial segmentation (S-1), decluster processing (S-2), and contour refinement (S-3).

**Figure 10:**
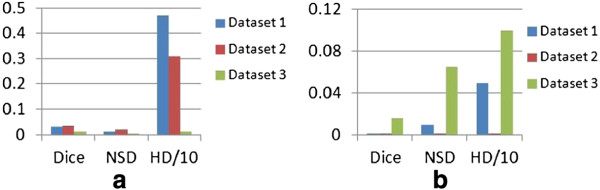
**Nucleus segmentation results.** Histograms with *x*-axis as the foreground probability derived from the intensity term,and *y*-axis as the numbers from per 100 pixels in $\bar{G}$ for **(a)** the real cell nuclei and **(b)** background.

**Figure 11:**
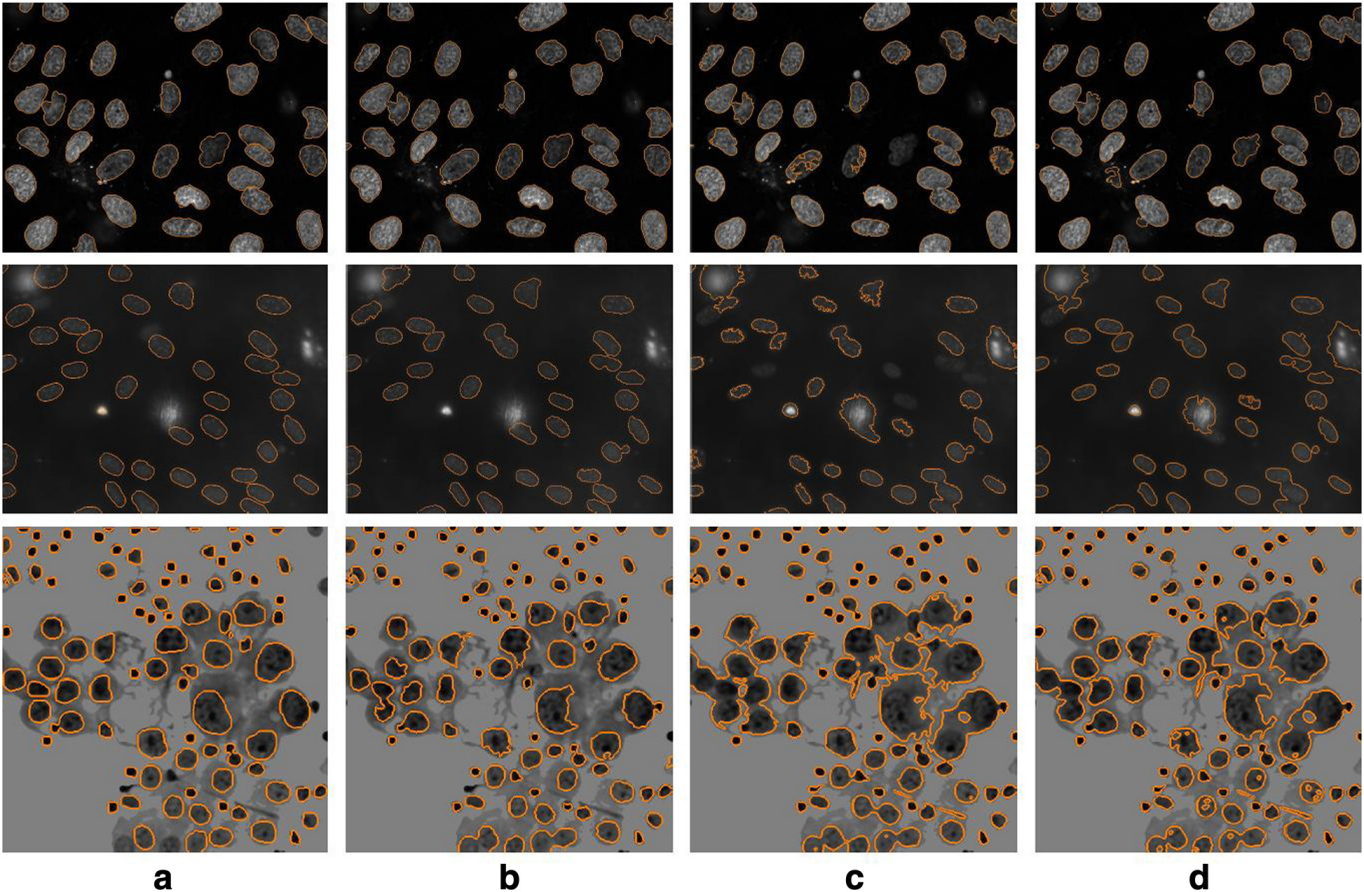
**Nucleus segmentation results.** Improvement on segmentation from **(a)** contrast-based detection term and **(b)** spatial term.

To further evaluate the design of the graphical model for contour refinement, the foreground probabilities for all pixels of interest are computed with the intensity term, as summarized in Figure 10. While many pixels exhibit suitable probabilities, some background pixels, especially those in datasets 2 and 3, have larger foreground probabilities and would lead to misclassification. The pixel-level classification is improved by introducing the contrast-based detection and spatial terms, as shown in Figure 11.

### Performance comparison

The localization results using the standard approaches are listed in Table 4, with example outputs shown in Figure 6. Compared to our proposed method, the level set and watershed techniques produce the second best results for dataset 1, especially with good contour-based measures. However, without explicitly handling high-intensity background regions, both methods result in about 3% lower detection precision. On dataset 2, our proposed method demonstrates stronger advantages, with 8.5% increase in detection accuracy,10.2% increase in Dice coefficient and 10.8 decrease in HD over the second best approach (i.e. level set). Both the level set and watershed approaches face the following challenges: (1) difficulty separating cell nuclei from surrounding background areas with low contrast, and (2) incapability of classifying background regions that resemble cell nuclei. On dataset 3, the intensity inhomogeneities within the cell nuclei and the cytoplasm make it particularly difficult to achieve good segmentation. As a result, the watershed method tends to largely over-segment the cell nuclei, generating many clusters and cause low detection recall and more errors in contour delineation. The level set method based on localized energy optimization is quite effective in splitting the clusters, but is less optimal for areas with high similarity between the cell nuclei and cytoplasm. The thresholding method does not perform as well as the level set or watershed approaches, but it does outperform the clustering-based approach. Compared to level set, our method achieves 7% increase in detection accuracy, 2.7% increase in Dice coefficient and 1.8 decrease in HD. Tables 2, 3 and 4 show that our proposed method delivers better localization even using only the initial segmentation step. Higher performance margins are obtained with decluster processing and contour refinement, especially on datasets 2 and 3.

**Table 4:** Comparison of localization results

	**Dataset 1**					
	**R**	**P**	**A**	**Dice**	**NSD**	**HD**
RPL	**0.975**	**0.940**	**0.916**	**0.958**	**0.017**	10.01
OT	0.928	0.807	0.767	0.876	0.068	17.52
KM	0.758	0.657	0.626	0.727	0.237	19.11
WS	0.974	0.915	0.893	0.945	0.022	11.21
LS	0.970	0.910	0.886	0.932	0.022	**9.58**
	**Dataset 2**					
	**R**	**P**	**A**	**Dice**	**NSD**	**HD**
RPL	**0.886**	**0.955**	**0.852**	**0.906**	**0.090**	**14.10**
OT	0.598	0.750	0.530	0.601	0.419	36.72
KM	0.508	0.601	0.437	0.521	0.503	134.5
WS	0.714	0.817	0.636	0.789	0.337	47.67
LS	0.832	0.899	0.767	0.804	0.207	24.96
	**Dataset 3**					
	**R**	**P**	**A**	**Dice**	**NSD**	**HD**
RPL	**0.957**	**0.975**	**0.934**	**0.924**	**0.052**	**5.51**
OT	0.712	0.961	0.685	0.873	0.188	31.80
KM	0.722	0.824	0.604	0.872	0.189	38.12
WS	0.584	0.989	0.575	0.819	0.335	34.53
LS	0.914	0.939	0.865	0.897	0.094	7.35

Comparison between our proposed region-based progressive localization method (RPL), Otsu thresholding (OT), k-means clustering (KM), watershed (WS) and level set [52] (LS).

A comparison with the state-of-the-art results reported for the same datasets is summarized in Table 5. Our method achieves better results in most measures, as bold-faced in the table. On dataset 1, 0.93 more FP cell nuclei are detected compared to the level set method [14]. It is possible that such false detections are caused by accidental highlighting of background regions during the iterative image enhancement for the initial segmentation stage. However, our method exhibits overall much better detection performance with minimal numbers of FNs (3.68 fewer than [14]) and only 1.43 FPs. The accuracy of pixel-level segmentation on dataset 2 improves significantly, as indicated by the 5% increase in Dice and Rand indices over [14]. 4.1% performance improvement of object-level accuracy over [35] on dataset 3 is also obtained. These observations suggest that our method is indeed quite effective in handling the intensity inhomogeneity issue that is the major cause hindering satisfactory segmentation on datasets 2 and 3. The improvement on the contour-based measures, i.e. on average 0.03 NSD decrease and 1.45 HD decrease over [14], also demonstrate the suitability of boundary delineation using region-based designs, i.e. the salient region extraction and graphical model-based contour refinement.

**Table 5:** Comparison with the state-of-the-art results

	**Dataset 1**					
	**Dice**	**Rand**	**NSD**	**HD**	**Add**	**Miss**
RPL	**0.958**	**0.959**	**0.017**	**10.01**	1.43	**0.12**
[14]	0.94	–	0.05	12.8	**0.5**	3.8
[38]	–	0.94	0.086	95.8	1.6	4.3
	**Dataset 2**					
	**Dice**	**Rand**	**NSD**	**HD**	**Add**	**Miss**
RPL	**0.906**	**0.932**	**0.090**	**14.10**	**1.47**	**1.17**
[14]	0.85	–	0.12	14.2	2.8	6.1
[38]	–	0.88	0.29	134.1	3.3	3.8
	**Dataset 3**					
	**Pix**	**Obj**				
RPL	**0.862**	**0.934**				
[32]	0.851	0.840				
[35]	0.856	0.893				

‘–’ means not available.

Our method is currently implemented in Matlab, running on a standard PC with a 2.66-GHz dual core CPU and 3.6 GB RAM. The computational time is related to the number of cells and the size of cells in an image. On a 1344 ×1024 pixel image with about 40 cell nuclei, an average 35 s is needed for the entire localization process. This is faster than applying the level set method [52], which requires about 45 s with 10 iterations.

## Conclusions

A fully automatic localization method for cell nuclei in microscopic images is presented in this paper. Intensity inhomogeneities in cell nuclei and the background often cause unsatisfactory localization performance. Not many works have been reported to address this problem in a robust manner. We propose a method that exploits various scales of data-adaptive information to tackle the intensity inhomogeneity. First, the regions of interest, i.e. cell nuclei or clusters, are extracted as salient regions with iterative contrast enhancement. Then with feature-based classification and reference-based probability inference, the clusters are further processed to detect more cell nuclei and filter out spurious regions. Lastly, based on regional contrast information encoded in a graphical model, the pixel-level segmentation is enhanced to create the final contours. This region-based progressive localization (RPL) method has been successfully applied to three publicly available datasets, showing good object-level detection and region- and contour-based segmentation results. Compared to popular approaches in this problem domain such as level sets, our method achieved consistently better performance, with on average 5.2% increase in Dice coefficient and 6% increase in object-level detection accuracy. Our method also outperformed the state-of-the-art with on average 3.5% and 7% improvement of region- and contour-based segmentation measures. We also suggest that the proposed method is general in nature and can be applied to other localization problems, as long as the objects of interest can be modeled as salient regions with measurable contrast from the background.

As a future study, we will investigate improving the graphical model for better contour delineation. A potential approach is to incorporate an additional term as the cost of difference between the model image and the measured image, as inspired by [24]. The model image could be derived as a convolution of a point-spread function of the microscope with an object intensity function defined based on the pixel labels. We will also investigate replacing the pixel-wise labeling with region-level processing for computational efficiency while maintaining the segmentation accuracy. Other future work could explore the applicability of the proposed method on other types of images. Images with nuclear membrane marker and different nuclear markers such as the green fluorescent protein (GFP), and those with higher resolution or dimension, are of particular interest. To accommodate the specific characteristics of these images, possible changes to the method are to design more comprehensive intensity and texture features to differentiate among cell structures and background, and to enhance the contour refinement with boundary constraints.

## Competing interests

The authors declare that they have no competing interests.

## Authors’ contributions

YS designed and carried out research and drafted the manuscript. WC discussed and helped to design the methodology and draft the manuscript. HH, YW and MC helped with the draft manuscript. DF coordinated the designed research. All authors read and approved the final manuscript.
